# Post-translational protein S-palmitoylation in renal tubular injury: mechanisms and therapeutic implications

**DOI:** 10.3389/fcell.2026.1960988

**Published:** 2026-09-01

**Authors:** Miao Li, Han Zhu, Huiping Zhao, Chenyang Liu, Yan Wang, Li Zuo

**Affiliations:** 1 Department of Nephrology, Peking University People’s Hospital, Beijing, China; 2 Jiangxi University of Chinese Medicine, Nanchang, Jiangxi, China; 3 Peking-Tsinghua Center for Life Sciences, Center for Quantitative Biology (CQB), Academy for Advanced Interdisciplinary Studies, Peking University, Beijing, China

**Keywords:** acute kidney injury, chronic kidney disease, post-translational modifications, renal tubular injury, S-palmitoylation, targeted therapy

## Abstract

Renal tubular injury contributes substantially to the progression of acute kidney injury (AKI) and chronic kidney disease (CKD). S-palmitoylation is a reversible lipid modification that regulates protein localization, stability, trafficking, and signaling. In renal tubular epithelial cells (TECs), it supports epithelial polarity, ciliary function, ion transport, and receptor activity. During renal tubular injury, dysregulated S-palmitoylation can disrupt tubular homeostasis by altering epithelial cell responses. It may aggravate cell damage, amplify inflammation, and promote failed repair and fibrosis. Changes in renal vascular cells and podocytes may also influence tubular injury through renal perfusion and the glomerular filtrate. However, the effects of S-palmitoylation are not the same. Some changes promote injury, whereas others preserve tubular function or limit injury signaling. The outcome depends on the modified protein, the enzyme involved, and the renal cell type. This mini-review summarizes the physiological roles of S-palmitoylation in renal tubules, its direct and indirect contributions to tubular injury, and therapeutic strategies targeting palmitoyltransferases, depalmitoylases, and specific substrates. Because global modulation may disrupt protective pathways, future interventions should prioritize pathogenic enzyme–substrate pairs and cell-specific delivery.

## 1 Introduction

Renal tubular injury is a common pathological feature of acute kidney injury (AKI) and chronic kidney disease (CKD). In AKI, the severity of tubular injury serves as a key determinant of adaptive versus impaired repair (Baker and Cantley, 2025), whereas in CKD, persistent or repeated tubular injury predominantly drives progressive tubular atrophy and tubulointerstitial fibrosis (Shao and Wei, 2025; Liao et al., 2026). Renal tubular epithelial cells (TECs), particularly those in the proximal tubule, rely on mitochondrial oxidative metabolism to sustain reabsorptive and secretory functions (Guo et al., 2025; Deng et al., 2025). This metabolic dependence makes them vulnerable to injury, which can impair epithelial transport and mitochondrial homeostasis and may result in tubular cell injury and trigger regulated cell death (Lai et al., 2024; Dai et al., 2026). During adaptive repair, surviving TECs dedifferentiate and proliferate to restore epithelial integrity (Kusaba et al., 2014). However, severe, persistent, or repeated injury can result in incomplete or maladaptive repair (Wang and Zhang, 2022; Li et al., 2024), including the persistence of failed repair proximal tubular cells with proinflammatory and profibrotic phenotypes (Gao et al., 2025; Wang et al., 2023b). Crosstalk with immune, endothelial, and interstitial cells further shapes the balance between recovery and chronic damage, ultimately promoting tubular atrophy, tubulointerstitial fibrosis, and the transition from AKI to CKD (Bao et al., 2024; Baker and Cantley, 2025).

Renal tubular injury is accompanied by remodeling of protein abundance and post-translational modifications, which can alter protein activity, stability, localization, interactions, and turnover (Zhong et al., 2023). Among these modifications, S-palmitoylation, also referred to as protein S-acylation, is a reversible lipid modification in which fatty acyl groups are attached to cysteine residues through thioester bonds (Mesquita et al., 2024; Qian et al., 2025). This process is primarily catalyzed by zinc finger DHHC-type palmitoyltransferases (ZDHHCs), with palmitoyl-CoA serving as the acyl donor, whereas depalmitoylation is mediated by acyl-protein thioesterases (APTs), palmitoyl protein thioesterases (PPTs), and α/β-hydrolase domain-containing proteins (ABHDs) (Yuan et al., 2024). Palmitoyl-CoA is sourced from *de novo* fatty acid synthesis and exogenous fatty acid uptake, making its cellular pool sensitive to metabolic flux—including dietary lipid intake and fatty acid oxidation—which can in turn affect S-palmitoylation dynamics (Xiu et al., 2025). Through this reversible cycle, S-palmitoylation regulates the membrane association, trafficking, stability, and interactions of substrate proteins (Shang et al., 2025; Xiu et al., 2025). Rapid palmitoylation turnover can further enable dynamic changes in protein localization and signaling. The thioester bond linking palmitate to cysteine is relatively labile, and depalmitoylation by APTs and ABHDs can occur on a timescale of minutes (Martin et al., 2011; Mesquita et al., 2024). This rapid reversibility allows S-palmitoylation to dynamically regulate protein membrane association and signaling in response to cellular stress, including in TECs during tubular injury.

In addition to its functions in normal protein regulation, aberrant S-palmitoylation contributes to disease-related damage in various cell types. Studies in other disease models have linked abnormal palmitoylation to mitochondrial dysfunction, oxidative injury, fibrosis, and pyroptosis (Li et al., 2023; Balasubramanian et al., 2024; Wang H. et al., 2024). In the kidney, this modification has been studied in both physiology and pathology. Importantly, its biological effects are highly dependent on the substrate modified, the enzyme responsible, and the renal cell type affected. This review summarizes the physiological functions of S-palmitoylation in the renal tubule, then discusses its pathological contributions to tubular injury and associated renal processes, and reviews current therapeutic strategies and future directions. We hope this review will serve as a useful reference for future experimental and clinical studies.

## 2 Physiological roles of S-palmitoylation in renal tubular structure and function

TECs maintain extracellular fluid volume, electrolyte composition, and acid–base balance through specific segment reabsorptive and secretory processes (Hoenig et al., 2025). These functions rely on mitochondrial oxidative metabolism, particularly in proximal TECs (Chen et al., 2023), and require the polarized organization of TECs and the accurate targeting and trafficking of membrane proteins among apical, basolateral, and specialized membrane domains (Brown, 2000).

Reversible S-palmitoylation regulates mitochondrial dynamics and function. Mitochondrial S-depalmitoylation is active and responds to changes in mitochondrial lipid homeostasis (Kathayat et al., 2018). At endoplasmic reticulum–mitochondria contact sites, palmitoylation of cytoskeleton-linking membrane protein 63 (CLIMP-63) at Cys100 is required for its interaction with voltage-dependent anion channel 2 and helps maintain mitochondrial membrane potential and respiration (Harada et al., 2020). ZDHHC13 palmitoylates dynamin-related protein 1 (DRP1), promoting its mitochondrial localization and normal fission–fusion and contributing to normal mitochondrial morphology and ATP production (Napoli et al., 2017).

S-palmitoylation can regulate polarized architecture. At tight junctions, palmitoylation of claudin-14 determines its efficient localization to tight junctions, but this modification does not affect protein stability and strand assembly (Van Itallie et al., 2005). In addition, ZDHHC5 and ZDHHC8 palmitoylate Ankyrin-G. This modification is required for its lateral membrane targeting, βII-spectrin recruitment, and lateral membrane assembly (He et al., 2012; 2014). ADP-ribosylation factor-like protein 13B (ARL13B) requires palmitoylation at Cys8 and Cys9 in mouse kidney to achieve ciliary localization and maintain normal ciliogenesis (Roy et al., 2017).

S-palmitoylation also directly regulates ion-channel activity involved in tubular ion transport. S-palmitoylation of the β and γ subunits enhances epithelial sodium channel (ENaC) activity by increasing channel open probability and reducing Na^+^-dependent self-inhibition (Mueller et al., 2010; Mukherjee et al., 2014). Multiple ZDHHC enzymes interact with ENaC and regulate its palmitoylation-dependent activation (Mukherjee et al., 2017), and mutation of these sites markedly reduces ENaC open probability in connecting tubules and cortical collecting ducts *in vivo* (Nickerson et al., 2023). For chloride transport, palmitoylation of barttin, the obligatory accessory subunit of chloride channel K (ClC-K) channels, increases the fraction of functional ClC-K/barttin channels at the plasma membrane but does not affect channel delivery to the membrane or conductance of individual channels (Steinke et al., 2015). ZDHHC7 is a major barttin palmitoyl acyltransferase in the kidney, and *ZDHHC7* deletion reduces renal barttin S-palmitoylation and produces hyponatremia and mild metabolic alkalosis during dietary salt restriction (Gorinski et al., 2020).

Beyond structural and transport functions, S-palmitoylation also regulates receptor signaling in TECs. Palmitoylation of the dopamine D1 receptor promotes its partitioning into lipid raft domains and maintains receptor-dependent cyclic adenosine monophosphate (cAMP) production (Tiu et al., 2020; Feng and Jin, 2025). The resulting cAMP signal inhibits sodium transport and promotes natriuresis (Tiu et al., 2020). Similarly, palmitoylation of the vasopressin V2 receptor regulates its surface expression, receptor endocytosis after agonist stimulation, and downstream extracellular signal-regulated kinase (ERK) signaling (Sadeghi et al., 1997; Charest and Bouvier, 2003).

Together, these findings show that S-palmitoylation supports the spatial organization and physiological regulation of TECs, whereas disruption of this dynamic balance may compromise tubular homeostasis during injury.

## 3 Dysregulated S-palmitoylation in renal tubular injury

**FIGURE 1:**
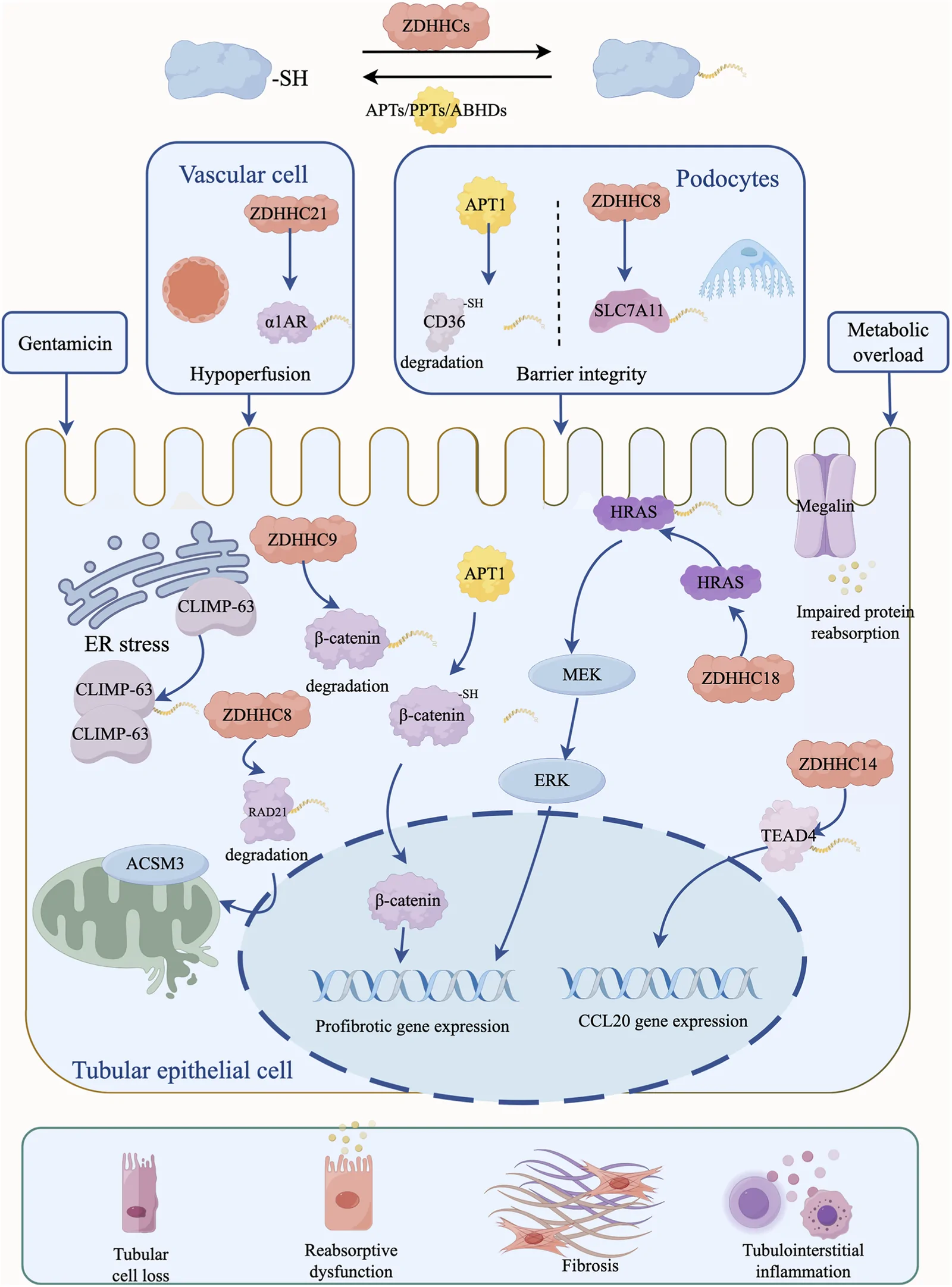
Overview of S-palmitoylation-mediated mechanisms in renal tubular injury (By Figdraw).

Renal tubular injury involves both autonomous responses within TECs and interactions with other renal cell populations (Scheidereit et al., 2026). Dysregulated S-palmitoylation may therefore act directly in TECs or indirectly through intrarenal crosstalk, as illustrated in Figure 1 and summarized in Table 1. Its effects are substrate specific and may be pathogenic or protective.

**TABLE 1:** Summary of S-palmitoylation mechanisms involved in renal tubular injury.

Injury context	Model	Regulator	Substrate (site)	Molecular mechanism	Effect on tubular injury	References
Nephrotoxic tubular injury	KPT11/KDT3 tubular cells	-	CLIMP-63 (Cys100)	Palmitoylation facilitates gentamicin-induced CLIMP-63 dimerization and caspase-3 activation	Pathogenic: promotes TEC apoptosis after gentamicin exposure	(Karasawa et al., 2010)
Lipotoxic tubular injury	HFD mice; HEK293 cells	-	Megalin (site unknown)	HFD reduces both S-acylated and total megalin. Global inhibition of S-acylation decreases megalin abundance and surface binding of gentamicin, which was used as a megalin ligand	Potentially pathogenic: may impair proximal-tubular protein reabsorption and contribute to albuminuria	(Xiu et al., 2025)
Diabetic tubular injury	Diabetic nephropathy mice; HK-2 cells	ZDHHC8	RAD21 (Cys78)	Promotes HSPA8-mediated lysosomal degradation of RAD21, repressing ACSM3 transcription and causing mitochondrial dysfunction	Pathogenic: aggravates tubular injury and mitochondrial dysfunction	(Chen et al., 2026)
Inflammatory amplification of tubular injury	Human IgAN kidneys; BSA nephritis mice; primary TECs	ZDHHC14	TEAD4 (Cys367 in human/Cys360 in mouse)	Enhances TEAD4 transcriptional activity and CCL20 expression	Pathogenic: promotes CCR6+ Th17 recruitment, tubulointerstitial inflammation, and fibrosis	(Zhou et al., 2025)
Maladaptive repair and fibrotic progression	Human CKD kidney; UUO/IRI mice; primary TECs	ZDHHC9	β-catenin (Cys300)	Palmitoylation promotes association with the destruction complex, polyubiquitination, and proteasomal degradation	Protective: limits β-catenin accumulation and profibrotic signaling	(Gu et al., 2023)
		APT1	β-catenin (Cys300)	Depalmitoylation inhibits β-catenin ubiquitination and degradation, thereby increasing its stability and nuclear accumulation	Pathogenic: sustains β-catenin signaling and promotes fibrosis	
	Human CKD kidney; UUO/FA mice; HK-2 cells and primary TECs	ZDHHC18	HRAS (Cys181/Cys184)	Promotes HRAS localization to the plasma membrane and MEK–ERK–RREB1 signaling	Pathogenic: promotes partial epithelial-to-mesenchymal transition, tubular atrophy, and fibrotic progression	(Lu et al., 2025)
Sepsis-associated renal hypoperfusion	Septic injury mice mouse/human renal arteries	ZDHHC21	α1AR (site not specified)	Enhances α1AR palmitoylation, ERK signaling, and renal vasoconstriction	Pathogenic: reduces renal perfusion and oxygenation and aggravates tubular injury	(Yang et al., 2021)
Podocyte lipotoxicity	db/db mice; human podocytes	APT1	CD36 (Cys466)	Depalmitoylation promotes lysosomal trafficking and degradation; loss of APT1 favors membrane recycling through Rab11	Protective: limits podocyte lipotoxicity and filtration-barrier injury and may reduce downstream tubular stress	(Wang et al., 2025)
	db/db mice; MPC5 podocytes	ZDHHC8	SLC7A11 (Cys327)	Palmitoylation limits ubiquitin-dependent turnover and preserves the cystine–GSH–GPX4 antioxidant pathway	Protective: maintains integrity of the filtration barrier and may reduce secondary tubular stress	(Yu et al., 2026)

Abbreviations: ACSM3, acyl-CoA synthetase medium-chain family member 3; APT1, acyl-protein thioesterase 1; BSA, bovine serum albumin; CCL20, C-C motif chemokine ligand 20; CCR6, C-C motif chemokine receptor 6; CD36, cluster of differentiation 36; CKD, chronic kidney disease; CLIMP-63, cytoskeleton-linking membrane protein 63; CLP, cecal ligation and puncture; Cys, cysteine; ERK, extracellular signal-regulated kinase; FA, folic acid; GPX4, glutathione peroxidase 4; GSH, glutathione; HFD, high-fat diet; HSPA8, heat shock protein family A member 8; IgAN, immunoglobulin A nephropathy; IRI, ischemia–reperfusion injury; MEK, mitogen-activated protein kinase kinase; RAD21, RAD21 cohesin complex component; RREB1, Ras-responsive element-binding protein 1; SLC7A11, solute carrier family 7 member 11; TECs, tubular epithelial cells; TEAD4, TEA domain transcription factor 4; Th17, T helper 17; UUO, unilateral ureteral obstruction; ZDHHC, zinc finger DHHC-type palmitoyltransferase; α1AR, α1-adrenergic receptor.

### 3.1 Direct effects on TECs

#### 3.1.1 Toxic tubular injury

Gentamicin nephrotoxicity primarily affects proximal TECs (Le et al., 2023). After tubular uptake, gentamicin accumulates in these cells and induces lysosomal phospholipid deposition, mitochondrial dysfunction, oxidative stress, and cell death (Le et al., 2023; Korkut Celikates et al., 2026). Karasawa et al. identified the endoplasmic reticulum-resident protein cytoskeleton-linking membrane protein 63 (CLIMP-63) as a gentamicin-binding protein and showed that its palmitoylation is required for dimerization after gentamicin exposure (Karasawa et al., 2010). Mutation of Cys100 reduced basal and gentamicin-induced dimer formation, whereas *CLIMP-63* siRNA suppressed caspase-3 activation and apoptosis. Although ZDHHC2 palmitoylates CLIMP-63 in nonrenal cells (Planey et al., 2009), the palmitoyltransferase responsible in tubular cells exposed to gentamicin remains unknown. Gentamicin-induced tubular injury also involves necroptosis mediated by receptor interacting serine/threonine protein kinase 1 (RIPK1)/RIPK3 signaling (Hu et al., 2022). Recent mechanistic studies further showed that ZDHHC5 palmitoylates RIPK1 and increases its kinase activity (Zhang et al., 2024), raising the possibility that RIPK1 palmitoylation may also contribute to gentamicin-induced tubular necroptosis. Identification of the responsible palmitoyltransferase–substrate pairs in tubular cell death may therefore provide a more selective therapeutic strategy than globally inhibiting protein palmitoylation.

#### 3.1.2 Metabolic tubular injury

Dysregulated glucose and lipid metabolism contributes to tubular injury through mitochondrial dysfunction and oxidative stress (Cai et al., 2025; Jiang et al., 2025; Sun et al., 2025). Diabetic tubulopathy is present from the early stages of hyperglycemia and contributes to diabetic kidney disease progression, which is influenced by multiple molecular pathways and genetic factors (Zhao et al., 2018; Liu et al., 2024; Zhang et al., 2025). Xiu et al. found that high-fat diet broadly reduced renal protein S-acylation across the proximal tubule, loop of Henle, and collecting system (Xiu et al., 2025). HFD increased ZDHHC9 and ZDHHC17 and decreased APT1 and APT2. Acyl-CoA synthetase family member 2 (ACSF2), which contributes to acyl-CoA synthesis, was reduced, suggesting that limited acyl-CoA availability contributed to the global hypo-acylation. Both S-acylated and total megalin were reduced, and pharmacological inhibition of S-acylation decreased megalin abundance and surface gentamicin binding. Considering that megalin mediates proximal-tubular retrieval of filtered proteins (Kulkarni and Hussain, 2025), these changes may decrease protein reabsorption and contribute to albuminuria. Chen et al. reported that ZDHHC8 palmitoylated cohesin complex component (RAD21) at Cys78, thereby promoting its heat shock protein family A member 8 (HSPA8)-mediated lysosomal degradation in diabetic nephropathy (Chen et al., 2026). Reduced RAD21 suppresses acyl-CoA synthetase medium-chain family member 3 (ACSM3) transcription and causes mitochondrial dysfunction. In high-glucose-treated proximal tubular epithelial HK-2 cells, *ZDHHC8* knockdown increased RAD21 abundance and reduced cell injury and mitochondrial dysfunction.

#### 3.1.3 Inflammatory amplification of tubular injury

Inflammation is a common feature of kidney disease (Brendolan et al., 2024), and injured TECs are not merely passive targets of inflammation; they can interact with immune cells and amplify tubulointerstitial inflammation (Wang et al., 2023a; Bai et al., 2024; Ruan et al., 2025). IgA nephropathy (IgAN) is initiated by the glomerular deposition of IgA-containing immune complexes, which triggers complement activation and inflammation and subsequently promotes tubular injury, interstitial inflammation, and fibrosis (Cheung et al., 2025). T helper 17 (Th17) cell-related immune responses contribute to renal inflammation and glomerulonephritis (Turner et al., 2010). Zhou et al. reported that S-palmitoylation in injured TECs promotes recruitment of immune cells in IgAN (Zhou et al., 2025). Under inflammatory conditions, ZDHHC14 was upregulated in damaged TECs and promoted TEA domain transcription factor 4 (TEAD4) palmitoylation, thereby enhancing TEAD4 transcriptional activity and C-C motif chemokine ligand 20 (CCL20) expression without altering its total protein abundance. Increased CCL20 subsequently recruited C-C motif chemokine receptor 6 (CCR6)-positive Th17 cells and aggravated tubulointerstitial inflammation. *Zdhhc14* knockdown or mutation of the TEAD4 palmitoylation site reduced CCL20 expression and Th17 migration. In IgAN model mice, 2-bromopalmitate (2-BP) also reduced Th17 infiltration and interstitial fibrosis. In addition to promoting immune cell recruitment, S-palmitoylation may amplify inflammation by regulating pyroptosis. Gasdermin D (GSDMD) mediates tubular pyroptosis and IL-18 release during AKI (Miao et al., 2019). Meanwhile, GSDMD palmitoylation promotes membrane translocation and pore formation, and inhibition of this modification suppresses macrophage pyroptosis and IL-1β release (Balasubramanian et al., 2024). Therefore, targeting pathological S-palmitoylation may limit tubular injury in inflammatory kidney diseases. However, TEAD4 is a transcriptional effector of Hippo signaling, and its palmitoylation supports protein stability and transcriptional activity (Noland et al., 2016). Because TEAD-dependent transcription contributes to tubular regeneration after AKI (Chen et al., 2018), excessive inhibition may compromise physiological repair. Therapeutic inhibition will therefore need to achieve selectivity for pathways and cell types.

#### 3.1.4 Maladaptive tubular repair and fibrotic progression

Persistent tubular injury can leave TECs in a state of failed repair, with sustained profibrotic signaling (Kirita et al., 2020; Wang J. et al., 2024; Wang and Wu, 2025). G2/M arrest and partial epithelial phenotypic change can reinforce this state and promote fibrosis (Yang et al., 2010; Lovisa et al., 2015). S-palmitoylation regulates this process in a manner that depends on the specific substrate (Lu et al., 2025; Gu et al., 2023). Gu et al. identified a protective palmitoylation pathway centered on β-catenin, a signaling effector whose sustained activation promotes renal fibrogenesis (Gu et al., 2023; Chen and Xue, 2024). ZDHHC9 palmitoylates β-catenin at Cys300 and promotes its polyubiquitination and proteasomal degradation, whereas APT1 removes the palmitoyl group, stabilizes β-catenin, and increases its nuclear accumulation. In human CKD kidneys and mouse models of unilateral ureteral obstruction (UUO) and post-ischemic fibrosis, ZDHHC9 expression and β-catenin palmitoylation were reduced, while APT1 was increased. Tubule-specific deletion of *Zdhhc9* aggravated β-catenin accumulation and fibrosis. By contrast, Lu et al. identified a pathogenic palmitoylation pathway centered on ZDHHC18 and HRas proto-oncogene, GTPase (HRAS) (Lu et al., 2025). In injured and failed-repair TECs, increased ZDHHC18 expression enhances HRAS palmitoylation at Cys181 and Cys184, promotes HRAS localization to the plasma membrane. This, in turn, enhances downstream MEK/ERK signaling and activates Ras-responsive element-binding protein 1 (RREB1), thereby amplifying profibrotic transcription in response to transforming growth factor β1. Deletion of Zdhhc18 specifically in TECs reduced tubular atrophy, epithelial phenotypic changes, inflammatory and profibrotic mediator expression, and matrix deposition in UUO and models induced by folic acid. These findings indicate that the consequences of S-palmitoylation in maladaptive tubular repair depend on the modified substrate rather than on a uniform increase or decrease in total protein palmitoylation.

### 3.2 S-palmitoylation-mediated intrarenal crosstalk in tubular injury

Renal tubular homeostasis depends on epithelial cell–autonomous responses, renal microvascular oxygen delivery, and the composition of the glomerular filtrate. Proximal tubules couple intensive solute transport to oxidative metabolism. Reduced regional oxygen delivery readily produces tubulointerstitial hypoxic stress (Palm and Nordquist, 2011). During septic injury, α1-adrenergic receptor (α1AR) palmitoylation increased in renal arteries. ZDHHC21 enhances α1AR palmitoylation, α1AR/ERK signaling, and renal vasoconstriction, reducing renal blood flow, tissue perfusion, and oxygen saturation. In contrast, loss of ZDHHC21 suppresses receptor palmitoylation and lessens brush border loss, epithelial detachment, and necrosis in proximal tubules (Yang et al., 2021). Even after blood pressure and cardiac output are restored, renal microvascular perfusion may remain impaired during sepsis (Harrois et al., 2018), and palmitoylation may contribute to this impairment. At the glomerular filtration barrier, S-palmitoylation regulates podocyte homeostasis by controlling the fate of membrane proteins. In diabetic podocytes, loss of cluster of differentiation 36 (CD36) depalmitoylation by APT1 redirects CD36 from lysosomal degradation to recycling through Rab11 GTPase. More CD36 then returns to the membrane and sustains fatty acid uptake and lipotoxic injury (Chen et al., 2024; Wang et al., 2025). By contrast, palmitoylation of solute carrier family 7 member 11 (SLC7A11) has a protective effect in podocytes. ZDHHC8 palmitoylates SLC7A11 at Cys327 and stabilizes this protein, which maintains glutathione (GSH) metabolism, reduces lipid peroxidation and ferroptosis, and preserves filtration barrier integrity (Yu et al., 2026). Meanwhile, SLC7A11 also protects against tubular ferroptosis in DKD (Lu et al., 2023), making its palmitoylation a potential regulatory mechanism in TECs. Loss of filtration-barrier selectivity increases the passage of albumin and other plasma proteins into the glomerular filtrate, thereby increasing the protein load delivered to proximal tubules. Sustained uptake of this protein overload disrupts lysosomal homeostasis and increases ROS production (Li et al., 2021; Eleftheriadis et al., 2023), and can activate inflammatory signaling and promote tubulointerstitial injury (Faivre et al., 2025; Fogo and Harris, 2025).

## 4 Therapeutic targeting of S-palmitoylation in renal tubular injury

The evidence for S-palmitoylation as a regulator of tubular injury has prompted interest in its therapeutic potential. Correcting aberrant S-palmitoylation may therefore limit tubular damage and improve renal outcomes.

### 4.1 Targeting palmitoyltransferases and depalmitoylases

The palmitoylation cycle can be targeted through inhibition of palmitoyltransferases or depalmitoylases. The broad inhibitor 2-BP has been widely used experimentally, but chemical proteomics shows that it reacts with multiple ZDHHCs and other proteins in lipid metabolism; this lack of selectivity limits its therapeutic value (Davda et al., 2013; Lan et al., 2021). More selective tools include ML348 and ML349, which preferentially inhibit APT1 and APT2, respectively (Won et al., 2016). In renal fibrosis models, ML348 preserved protective β-catenin palmitoylation and reduced tubular injury and fibrosis (Gu et al., 2023). Outside kidney disease, ABD957 selectively inhibits ABHD17-family depalmitoylases and alters NRAS proto-oncogene, GTPase (NRAS) palmitoylation and signaling, showing that this enzyme class is pharmacologically tractable (Remsberg et al., 2021). Selective ZDHHC inhibitors remain less developed because of conserved catalytic architecture and overlapping substrate repertoires (Lan et al., 2021). For example, HRAS can be palmitoylated by multiple ZDHHCs, whereas ZDHHC8 targets distinct substrates, including RAD21 and SLC7A11 (Lu et al., 2025; Chen et al., 2026; Yu et al., 2026). Similarly, in cancer cells, artemisinin covalently binds ZDHHC6 and suppresses NRAS palmitoylation. This finding shows that specific isoform inhibition is feasible (Qiu et al., 2022). These findings indicate that palmitoylation and depalmitoylation enzymes are pharmacologically tractable. However, applying this approach to the kidney will require selectivity at the enzyme, substrate, and cell levels.

### 4.2 Strategies directed at specific substrates

The mammalian ZDHHC family comprises 23 isoforms with partially overlapping substrate repertoires (Lan et al., 2021). A single substrate can be palmitoylated by multiple ZDHHCs, as exemplified by HRAS (Lu et al., 2025), which is modified by several family members. Conversely, a single ZDHHC can act on distinct substrates with divergent biological functions—ZDHHC8, for instance, palmitoylates RAD21 to promote mitochondrial injury in TECs (Chen et al., 2026) yet palmitoylates SLC7A11 to preserve podocyte integrity (Yu et al., 2026). This catalytic redundancy implies that genetic deletion or pharmacological inhibition of one ZDHHC isoform may be partially compensated by others, potentially limiting therapeutic efficacy while still disrupting unrelated physiological pathways. Because individual substrates can have different biological effects, substrate-directed strategies may provide greater precision by interfering with a palmitoylation-dependent structural feature or functional property of a specific pathogenic protein without broadly altering the corresponding enzymatic machinery. Small molecules that occupy the internal pocket in TEAD proteins where lipids bind can disrupt transcriptional activity that depends on lipidation without directly inhibiting palmitoyltransferases (Holden et al., 2020). The peptide PTG-101 binds Ras-related protein Rap-2b (Rap2b) and masks the region containing cysteine residues, thereby reducing Rap2b palmitoylation, localization to the plasma membrane, and pathological activity in colorectal cancer models (Zhu et al., 2024). Engineered depalmitoylases have also enabled temporally and spatially controlled removal of palmitoylation from selected proteins or protein complexes (Jayaraman et al., 2025). However, these systems remain experimental, and their delivery and therapeutic efficacy in renal tubular injury remain untested. Studies in other systems show that the structure and location of a substrate can improve treatment selectivity. In principle, this strategy could suppress a pathogenic renal substrate while preserving other proteins regulated by the same enzyme.

### 4.3 Approaches based on metabolism, natural compounds, and drug repurposing

Beyond direct enzyme inhibition and substrate-directed intervention, changes in lipid metabolism, acyl donor availability, membrane composition, upstream signaling, or expression of proteins may indirectly reshape S-palmitoylation. The broad alterations in renal S-acylation observed under lipid overload indicate that reversible cysteine acylation in the kidney is sensitive to metabolic conditions (Xiu et al., 2025). Yitangkang, a traditional Chinese medicine formulation, influenced a protective palmitoylation pathway, and iproniazid restored a protective tubular pathway by increasing ZDHHC9 expression (Gu et al., 2023; Yu et al., 2026). Studies in other systems show that established metabolic drugs can alter protein palmitoylation without directly targeting the palmitoylation machinery. Metformin reduced protein kinase B (AKT) palmitoylation and membrane recruitment in macrophages, while simvastatin reduced caveolin-1 palmitoylation and caveolar localization in human leiomyoma models (Xiong et al., 2021; Afrin et al., 2022). These findings suggest that natural compounds and drug repurposing could also modify specific palmitoylation pathways in disease. However, whether metformin, simvastatin, and similar interventions alter S-palmitoylation in renal tubular injury remains unclear. Direct measurements of palmitoylation in injured tubular cells will be needed to determine whether the protective effects depend on these modifications.

## 5 Conclusion and future perspectives

S-palmitoylation participates in renal tubular injury and repair. Its effects depend on the protein, the enzyme, and the cell type, and can be protective or pathogenic. These findings suggest that indiscriminate global manipulation is not appropriate; instead, intervention should target specific enzymes or proteins.

A major limitation to therapeutic translation is the broad physiological roles of S-palmitoylation and the lack of selectivity for particular cell types. Delivery directed to the kidney may improve the therapeutic window (Xue et al., 2024). Nanoparticle systems that preferentially accumulate in renal tubules can reduce tubular cell injury in AKI models (Williams et al., 2018; Funahashi et al., 2024). Such carriers could be adapted to deliver small molecules, RNA therapeutics, competitive peptides, or engineered protein modulators while reducing systemic exposure. Targets in podocytes, renal vascular cells, or other components of the tubular microenvironment require different delivery properties. Target discovery and treatment monitoring depend on methods that distinguish changes in protein palmitoylation from changes in total protein abundance. Palmitoyl-proteomics can identify dynamically modified substrates (Martin and Cravatt, 2009). Integration with single-cell and spatial profiling could define the renal cell types and tissue regions in which these changes occur (Abedini et al., 2024). Human kidney atlases provide a framework for mapping these events to tubular states associated with injury and multicellular fibrotic niches (Lake et al., 2023). Translation will also require validating target engagement in human kidney tissue, renal organoids, and multicellular models, and developing biomarkers for specific substrates to aid dose selection and patient stratification.
